# Formulation of a Bio-Packaging Based on Pure Cellulose Coupled with Cellulose Acetate Treated with Active Coating: Evaluation of Shelf Life of Pasta Ready to Eat

**DOI:** 10.3390/foods9101414

**Published:** 2020-10-07

**Authors:** Valeria Bugatti, Gianluca Viscusi, Giuliana Gorrasi

**Affiliations:** 1 Department of Industrial Engineering, University of Salerno, via Giovanni Paolo II 132, 84084 Fisciano, Italy; vbugatti@unisa.it (V.B.); gviscusi@unisa.it (G.V.); 2 Nice Filler s.r.l., via Loggia dei Pisani 25, 80133 Napoli, Italy

**Keywords:** bio-packaging, pasta, layered double hydroxide, active coating

## Abstract

An active packaging based on pure cellulose coupled with cellulose acetate coated with layered double hydroxide (LDH), hosting 4-hydroxybenzoate (listed in EC-Directive 10/2011) as an antimicrobial agent, was formulated and realized. The release of 4-hydroxybenzoate ionically bonded to the LDH layers was much slower than the molecule freely dispersed into the coating. The capability of the active packaging to inhibit *Pseudomonas*, *Escherichia coli*, *Salmonella* and Lactic Bacteria was evaluated, as well as the global migration with three different food simulant (i.e., acetic acid at 3% (*v*/*v*), ethanol at 50% (*v*/*v*) and vegetable oil) that demonstrated, in compliance with the migration limits of the EU regulation, the suitability of the prepared packaging to be employed as food contact material. Ready to east cooked tomato pasta was packaged into the active trays and in uncoated, as control, up to 30 days at 4 °C. Organoleptic characteristics, mold evolution, total mesophilic aerobic counts (TBC), *Enterobacteriaceae*, Lactic Bacteria and *Pseudomonas*, and in colony forming unit per gram (CFU/g), showed a significant activity of 4-hydroxybenzoate in increasing the shelf life of the pasta ready to eat.

## 1. Introduction

In the global food industry, takeaway food is one of the most growing sectors. It is linked with increased continuous demand for packaging, the majority of which is single use, to satisfy hygienic issues and food safety. Food packaging materials are almost 26% of all plastics produced worldwide, and about 90% becomes waste after only one use [1]. Furthermore, single-use plastics are non-recyclable materials [2], and this represents a true environmental problem. As demand for packaging increases, the development of better options for food packaging that is decoupled from the use of limited resources, becomes ever more important. Based on circular economy principles, food packaging materials should be reusable, recyclable, or compostable by design and fit within solutions that carry this out in practice and on an industrial scale [3]. Pasta is the most known and appreciated Italian food worldwide. Its consumption has largely increased in the past decade, from 7 to 12 million tons/year, due to the largely offered pasta meals in fast food and international markets. Ready to eat pasta, in contrast to frozen one, generally is more convenient for reduced preparation times and by selecting takeaway packaging. The most common ready to eat pasta is generally composed of pasta and sauce (the most commonly used is tomato sauce). Two mixed physical phases with very different characteristics make pasta with tomato sauce ready to eat an extremely dynamic system subjected to several modifications during the storage [4,5].

In order to extend the shelf life of the packaged pasta as much as possible and to control microbial growth, an active packaging solution is the most promising. It has been demonstrated that the simple blend of antimicrobials into the food formulation is not the best choice because they are often neutralized in reactions with the food during storage [5]. Active packaging solutions are a valid alternative to overcome such limitations since they allow the controlled release of antimicrobial agents during storage and maintain their critical concentration necessary to inhibit the microbial growth [6,7]. Several strategies to achieve active molecules’ control release and modeling the transport phenomena have been reported [8,9,10,11,12,13]. Due to the importance of the topic, studies on the development of food packaging (composites and bio-composites) films with controlled release properties are increasing more and more [14,15,16,17,18,19,20,21,22,23,24,25,26,27,28,29]. The present paper reports the preparation of active trays based on compostable pure cellulose coupled with biodegradable cellulose acetate, as support for an active coating. The coating was realized using a food-grade resin in which was dispersed 4-hydroxybenzoate (listed in EC-Directive 10/2011) as an antimicrobial agent. Such active molecules have been previously ionically bonded to layered double hydroxides (LDH), an anionic natural clay, as a nano-carrier. The structural and thermal characterization of the active filler, as well as the study of the inhibition controlled release of 4-hydroxybenzoate was conducted. The in-vitro antimicrobial of the active trays was evaluated against *Pseudomonas, Escherichia coli, Salmonella* and Lactic Bacteria were evaluated, and the global migration with three different food simulants. Fresh cooked pasta with tomato, ready to eat, was packaged into the coated trays and uncoated, as control. The evaluation of the organoleptic characteristics, the evolution of *molds, TBC,* and *Enterobacteriaceae,* Lactic Bacteria and *Pseudomonas*, in CFU/g, was followed up 30 days of storage at 4 °C, highlighting a significant effect of LDH-(4-hydroxybenzoate) in improving the shelf life of the pasta meal.

## 2. Materials and Methods

### 2.1. Materials

Cellulose acetate (AC311025) used for the paper trays was supplied by Emilplast srl. (Modena, Italy) in film form, 25 μm thick. Cellulose acetate film was coupled to pure virgin cellulose cardboard in the form of trays for food. The active filler, having the trade name of N6B6^®^ is based on an LDH intercalated with antimicrobial 4-hydroxybenzoate anion, listed in EC-Directive 10/2011. It was produced by Nicefiller Ltd., a startup of the University of Salerno (Italy). The synthesis was conducted accordingly to a previously reported procedure [30]. The active molecule in the nano-hybrid N6B6^®^ is equal to 36 weight percentage (wt%).

The coating was done using a water-based resin for food packaging (Colorgraf 359509, solid content 40 ± 2%, viscosity 40 s at 20 °C) purchased from Colorgraf spa (Lainate, Italy). Its constituents are in accordance with the EC-Directive 2002/72, including amendments. The food-grade resin and the active filler at 10 wt% were mixed using high energy ball milling at ambient temperature, for 30 min at 450 rpm and coated on cellulose acetate by using an automatic coater. The cellulose acetate treated by coating with the active filler has been coupled to pure virgin cellulose cardboard to make paper trays for food (named active tray). Control trays based on the same component (i.e., cellulose coupled with cellulose acetate with no coating) were prepared and used as control (named uncoated tray). Freshly cooked pasta with tomato sauce was packaged (≅100 g) either in the active trays or in the uncoated and closed in an untreated cellulose acetate flowpack.

### 2.2. Methods

#### 2.2.1. Analysis of Active Filler and Packaging

X-ray diffraction (XRD) patterns were taken in reflection with an automatic Bruker diffractometer D8 (Karlsruhe, Germany), using nickel-filtered Cu Kα radiation (Kα = 1.54050 Å) and operating at 40 kV and 40 mA, with a step scan of 0.05° of 2ϑ and 3 sec of counting time.

Thermogravimetric analyses (TGA) were carried out in air atmosphere with a Mettler TC-10 thermo-balance (Mettler-Toledo GmbH, Greifensee, Switzerland) from 30 to 700 °C, in airflow, at a heating rate of 10 °C/min.

The release kinetics of the active molecule 4-hydroxybenzoate, which is a chromophore, were followed using a Shimadzu UV-2401 PC spectrometer (Shimadzu, Kyoto, Japan). The considered band was 246 nm, highlighted by the calibration line in physiological solution. The tests were performed using 4 cm^2^ square samples of packaging material placed into 25 mL physiological solution (NaCl 0.9%) and stirred, at room temperature, at 100 rpm in an orbital shaker (VDRL MOD. 711+, Asal S.r.l., Milan, Italy) for 30 days. The release medium (25 mL physiological solution) was withdrawn at fixed time intervals and replenished with fresh medium. The physiological solution withdrawn at every time, containing the active molecule, is analyzed at UV, and the absorbance corresponding to 246 nm is measured. From the calibration line, the concentration and, consequently, the percentage released is determined, which is reported as a function of time considering the packaging containing the active molecule anchored to the hydrotalcite compared with the packaging containing the free molecule.

The in-vitro effect of inhibition against *Pseudomonas, Escherichia coli, Salmonella,* and Lactic Bacteria by the active trays were analyzed following the directive ISO 22196:2011: such method evaluates the antibacterial activity of treated plastics, surfaces, and other non-porous materials. The test microorganism was prepared by growth in a liquid culture medium. The suspension of the test microorganism was standardized by dilution in a nutritive broth, then control and test surfaces were inoculated with microorganisms, in triplicate, and the microbial inoculum was covered with a sterile film. In order to provide a comparison, all microbiological assay runs were performed with the necessary parallel controls for the whole time of the experiments. Microbial concentrations were determined at “time zero” by elution, followed by dilution and plating. A control was run to verify that the neutralization/elution method effectively neutralized the antimicrobial agents in the antimicrobial surface being tested. The inoculated, covered control and antimicrobial test surfaces were allowed to incubate undisturbed in a humid environment for 24 h. After incubation, microbial concentrations were determined. The reduction of microorganisms relative to initial concentrations and the control surface was calculated. By including the proper controls and being able to make these reduction calculations, such assay allowed to evaluate whether the treated tray is bacteriostatic, having the ability to inhibit the growth of microorganisms. The procedural steps consisted of preparing the microbial suspension to be used as an inoculum; inoculation of microorganisms was in triplicate, both in untreated and in treated specimens, and covering with a sterile film. Incubation took place at 35 °C for 24 h in humid conditions; the microbial count present on the surface of the different treated and control specimens were determined after incubation by washing with a neutralizer (10 mL) and serial dilutions for the plate count with PCA medium. Microorganisms used were those provided by the official method ISO 22196: 2011. The size of the inoculum was between 2.5 × 105 and 10 × 105 CFU/mL. The effectiveness was measured by comparing the survival of the bacteria placed in contact with the treated and untreated materials. The various bacteria were grown in the nutrient broth (PBS). An aliquot of this culture was placed in contact with at least three treated and untreated samples. The samples were divided into portions of 50 × 50 mm size, inoculated with bacterial culture, and covered with a sterile film of 40 × 40 mm size. The colony-forming units were enumerated with a microbiological counting technique, and the antibacterial activity R was calculated from the results of the microbial count, which represented the elimination capacity on a logarithmic basis over a period of 24 h of the bacteria being in contact with the treated material. R = (Ut − U_0_) − (At − U_0_). The higher the R, the more the treated material has the ability to kill bacteria. The units of measurement of U_0_, Ut, and At are Log (CFU/cm^2^).

Overall migration tests were performed on the paper active trays according to the following procedure: film specimens with 1 dm^2^ of surface area (10 cm × 10 cm, 0.10 mm thickness) were put into contact with 100 mL simulant (preconditioned at 40 °C) in a borosilicate glass tube closed with a screw cap internally layered with Teflon^®^. The obtained surface/volume ratio was 10 dm^2^/L. Migration tests after contact for 10 days at 40 °C were performed using as simulants D2 (Vegetable oil), D1 (Ethanol at 50%) and B (Acetic acid at 3%). The overall migration limits applies to the sum of all substances that can migrate from the food contact material to the food simulant. The overall migration test was performed on different aliquots from the same contact sample. The overall migration results were calculated by using 6 dm^2^/kg food (6 dm^2^/L simulant) as a conventional EU surface/volume ratio. A known aliquot of the simulant from the contact solution was transferred into a weighted quartz capsule and evaporated to dryness until constant weight. From the differences between the weights, the overall migration was derived in accordance with EN 1186 Migration Testing for Food Contact Materials. The data were averaged on five samples.

Standard deviations of Figures 3–5 were calculated using the classical equation:

$$\sqrt{\frac{\sum {\left(x-x_{a}\right)}^{2}}{\left(n-1\right)}}$$

where *x* represents the value obtained from each experiment, *x_a_* the medium value, and *n* the number of experiments conducted.

#### 2.2.2. Analysis of Packaged Pasta

Organoleptic properties (appearance, color, texture, smell, and taste) have been analyzed according to the regulation UNI–U590B2560. Food sensory evaluation ensured that products and packaging were free from defects and/or contamination, and also measured and interpreted human responses based upon sight, smell, touch, taste, and hearing. This is an important aspect of product development and marketing since it offers insight into consumer behavior and quality assurance. For the sensory analyses, the pasta samples were cooked with water and salt for 20 min, and afterward, tomato sauce was added. The sensory analysis was conducted with ten non-trained academic tasters from the University of Salerno. The samples (around 25 g) were offered to the tasters on a disposable plastic plate, and each sample was identified by three random numbers. A water bottle was offered with the samples to remove any residual flavor from the mouth. Appearance, aroma, flavor, texture, and overall impression were evaluated using a 9-point hedonic scale with a minimum of 1 (extremely disliked) and a maximum of 9 (extremely liked) [31,32]

Microbiological tests on freshly cooked pasta, either packaged in active trays or in trays with no treatment, were conducted with the aim to measure: total mesophilic aerobic count (TBC) (ISO 4833:2003) [33], molds [34] Enterobacteriacae (AFNOR AES 10/07-01/08), Lactic Bacteria (ISO 15214:1998) [35], and Pseudomonas (ISO 13720:2010). Samples were divided into small pieces, placed in the trays with and without active filler and closed in an untreated cellulose acetate flowpack (165 × 135 × 45 mm). A total of 24 trays (3 replicates for 2 packages) for four storage times 0, 10, 20, and 30 days were stored at 4 °C for 30 days. At each storage time, samples were analyzed to follow the incidence of visible molds on the pasta, by analyzing the organoleptic properties. Mold evolution on pasta was evaluated as follows: ten grams of pasta sample were, in triplicate, aseptically homogenized in 90 mL of sterile 0.9% NaCl solution in a stomacher (Lab-Blender 400, PBI International, Milano, Italy), and decimally diluted in 0.1 % (*w*/*v*) sterile peptone saline solution (0.9 % NaCl) before plating on the selective media. In particular, molds were detected on potato dextrose agar (PDA) supplemented with 100 mg/L of chloramphenicol and incubated at 25 °C for 3–5 days (ISO 21527-1:2008). Total mesophilic aerobic counts (TBC) were enumerated on plate count agar (PCA) supplemented with 100 mg/L of cycloheximide after incubation at 30 °C for 24 h (ISO 4833:2003). Pseudomonas were grown on Pseudomonas agar base (PSA, amended with Pseudomonas CFC selective supplement, Oxoid) at 30 °C for 24 h (ISO 13720:2010); Lactic acid bacteria population (LABs) were counted on De Man, Rogosa, and Sharpe agar (MRS) supplemented with 100 mg/L of cycloheximide after incubation at 30 °C under anaerobic conditions for 48 h (ISO 15214:1998). Enterobacteriaceae were evaluated by RAPID’Enterobacteriaceae medium, high-performance medium for the enumeration of Enterobacteriaceae in food and environmental samples at 30 °C for 24 h (according to AFNOR AES 10/07-01/08). Microbiological values reported as (Log(CFU/g) were the average of three replicates.

Statistical analyses were conducted using Origin Lab. Results were expressed as the mean value ± standard deviation (SD). ANOVA and Tukey’s tests were performed to compare the obtained results at a significance level of α < 5%. The curve fitting for the Gompertz equation (Equation (1)) was evaluated by non-linear regression. The coefficient of determination (*R*^2^) was evaluated to assess the goodness of the fitting.

## 3. Results and Discussion

### 3.1. Analysis of Active Filler and Trays

**Figure 1:**
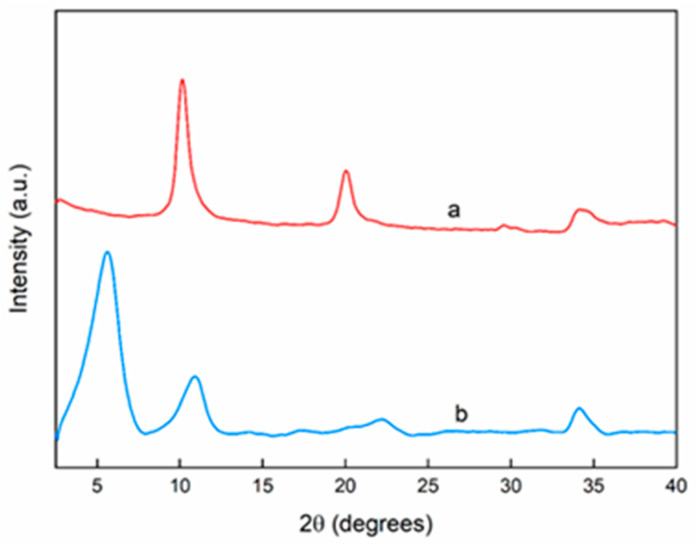
XRD of pristine LDH in nitrate form (**a**) and LDH hosting 4-hydroxybenzoate (**b**).

Figure 1 shows the LDH in nitrate form (a), with the peak characteristic of the basal spacing at 2ϑ = 10.2°, corresponding to an interlayer distance d = 8.6 Å. The intercalation of the 4-hydroxybenzoate anion into LDH galleries occurred successfully (Figure 1b), and this was demonstrated by a shift of the peak toward lower angle, 2ϑ = 5.6°, that corresponded to an increased interlayer distance (d = 14.86 Å) having the 4-hydroxybenzoate anion a steric hindrance higher than nitrate.

**Figure 2:**
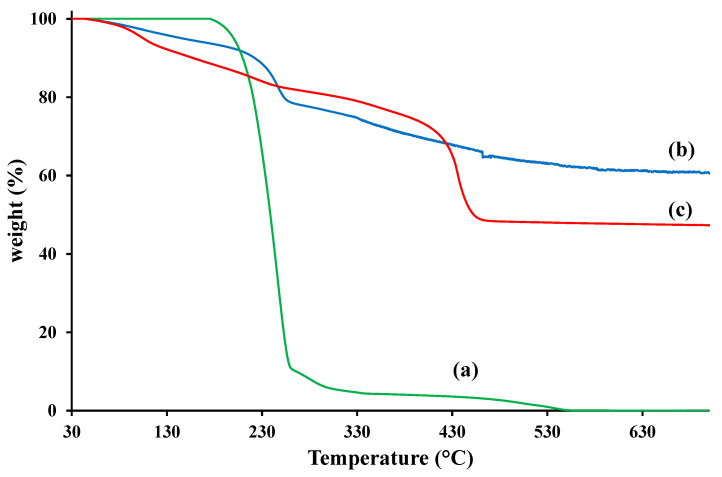
TGA analysis of 4-hydroxybenzoic acid (**a**), LDH-NO_3_ (**b**), and LDH-(4-hydroxybenzoate) (**c**).

Figure 2 reports the TGA analysis on of 4-hydroxybenzoic acid (a), LDH-NO_3_ (b), and LDH-(4-hydroxybenzoate) (c) in airflow. LDH with intercalated nitrate anion shows three main degradation steps [36]: the loss of absorbed water between the LDH galleries at about 150 °C, the thermal decomposition of nitrate anions at around 250 °C, the dehydroxylation of the LDH layers after 400 °C. It is evident that the 4-hydroxybenzoic acid degrades in one main step, centered at about 238 °C. The thermal stability of 4-hydroxybenzoate intercalated between LDH’s sheets results improved; in fact, the main decomposition of the nano-hybrid was centered at about 434 °C. Such behavior has already been found for several organic molecules intercalated into LDH layers [37], allows us to hypothesize a protecting effect from the inorganic LDH nano-carrier also for 4-hydroxybenzoate.

The release of 4-hydroxybenzoate (wt%) of the active molecule was evaluated as a function of time (days) from the active tray. A sample in which the active molecule was simply dispersed into the food-grade resin at the same percentage (i.e., 3.6%) and coated on the cellulose acetate was also prepared, following the same experimental conditions reported in Section 2.1.

**Figure 3:**
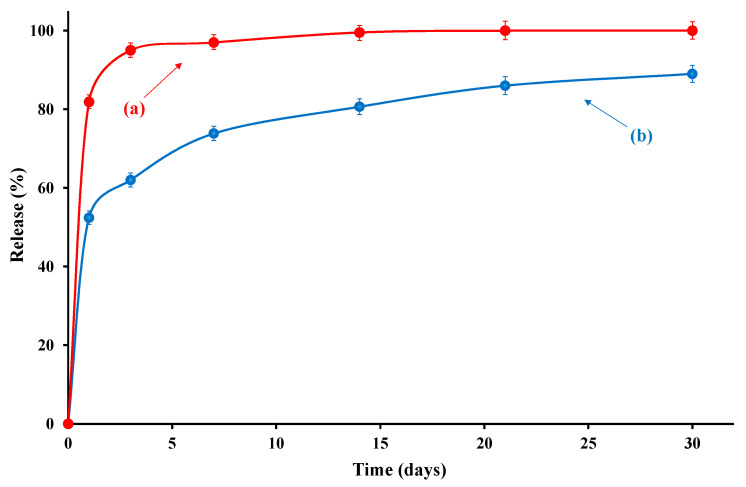
Release of 4-hydroxybenzoate (wt%) from: (**a**) cellulose acetate coated with 3.6 wt% of 4-hydroxybenzoate simply dispersed into the food-grade resin, and (**b**) cellulose acetate coated with active molecule anchored to LDH.

The release of 4-hydroxybenzoate freely dispersed into the coating medium was very fast and completed in almost 3 days (Figure 3). The release mode of the active molecule anchored to the LDH layers showed a first fast step, corresponding to the release from the surface of the material, followed by a second step related to the de-intercalation due to ionic exchange with chloride in physiological solution. It is evident that in the whole investigated range of time, the 4-hydroxybenzoate inside the active tray showed a slower release compared to the same molecule freely dispersed into the coating. In addition, in the considered range of contact time (i.e., 30 days) the molecule bonded to the nano-carrier was not completely released. This result was in agreement with XRD analysis that demonstrated the successful intercalation of the sorbate between the LDH galleries.

*Pseudomonas*, *Escherichia coli*, *Salmonella*, and *Lactobacillus* strains were used to test the bacterial inhibition capability from the active tray filled with LDH-(4-hydroxybenzoate). It is evident from Table 1 that the prepared active trays exerted a significant antibacterial activity with respect to all considered strains. The degree of inhibition of the 4-hydroxybenzoate molecule present in the active trays towards the bacteria analyzed ranged from 1 to 5 orders of magnitude higher than the untreated ones, highlighting a significant inhibition towards *Pseudomonas* and *Salmonella*; with respect to *Escherichia Coli* and *Lactobacillus*, even if slightly lower, there was still evidence of a bacteriostatic activity in the treated tray. The importance of the capability from the active packaging to have such important bacteria inhibition, common in food spoilage, allowed us to use that for storage of cooked tomato pasta. Although a certain cause and effect correlation for the mechanisms of action of parabens has not yet been established, several studies showed that such compounds could be active at the cytoplasmic membrane and capable of inhibiting both membrane transport and the electron transport system [38].

In order to demonstrate that the prepared active packaging is suitable for food contact, we performed overall migration tests on the active trays. Table 2 reports the overall migration, evaluated on the active trays, in different food simulants, according to UNI EN 1186-1:2003 and UNI EN 1186-9: 2003 simulant D2 (Vegetable oil), D1 (Ethanol at 50%), and B (Acetic acid at 3%). The experimental results, in compliance with the migration limits, demonstrate the suitability of the considered material for food contact. The prepared packaging, although new, can be easily used for food contact, due to the respected security level to preserve consumer health.

### 3.2. Evaluation of Shelf Life on Packaged Pasta Ready to Eat

Cooked pasta with tomato sauce (≅100 g), ready to eat, was packaged into the active trays and uncoated. Organoleptic properties, *Moulds*, *Total mesophilic aerobic counts* (TBC), *Enterobacteriaceae,* Lactic Bacteria and *Pseudomonas* were evaluated up to 30 days of storage at 4 °C. Table 3 below shows the organoleptic properties after 30 days of storage.

**Figure 4:**
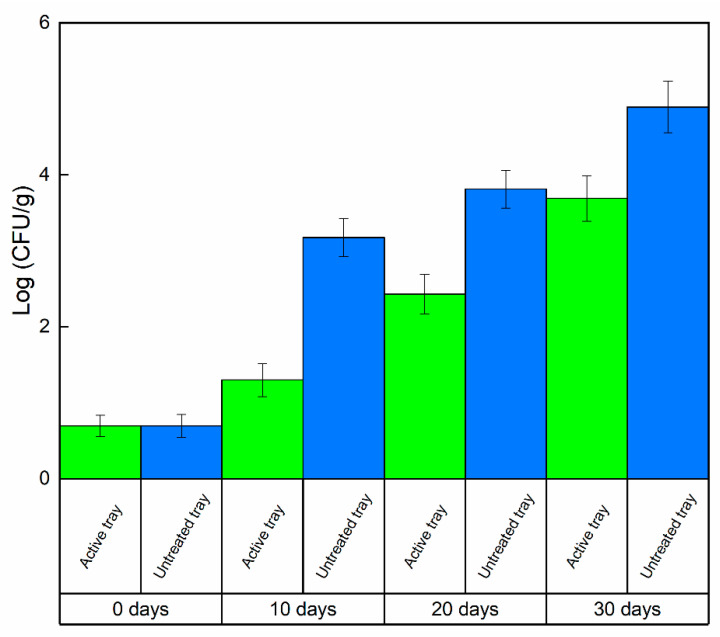
Mold evolution, Log (CFU/g) on packaged pasta @4 °C as a function of time.

**Figure 5:**
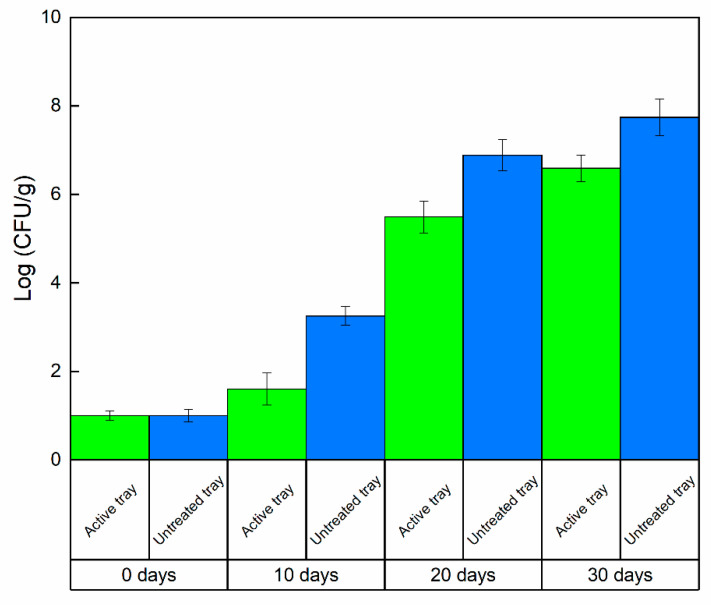
TBC evolution, Log (CFU/g) on packaged pasta @4 °C as a function of time.

The incidence molds on the surface of the cooked pasta, was evaluated by counting the number of pieces of pasta with visible mold on each bag for each replicate in the untreated packaging and in the active packages, expressed as a percentage of infected pieces in respect to the total. It was evident that the pasta packaged in uncoated trays and the active ones showed several differences in terms of appearance, smell, taste, and color. In addition, the presence of visible molds on the surface was significantly different (*p* <0.05): pasta stored in an uncoated tray was covered by ≅80% of mold on the surface, while pasta packaged in the active tray shows only ≅5% of visible molds. The presence of the active molecule on the surface of the bio-packaging has an antimicrobial and anti-mold effect; the anti-mold and antimicrobial activity of the molecule were known, but the hydrotalcite system modified with the organic molecule means that the release of the molecule occurred faster in the first hours and then more slowly over 30 days to ensure bacteriostatic activity on molds and bacteria both in the first few hours to block the proliferation of microbes, but also during the whole investigated storage time to ensure the maintenance of quality, food safety and freshness of the pasta for longer times. Figure 4 and Figure 5 report the mold and TBC evolution, respectively, as a function of the time (days), evaluated on pasta packaged either in the active tray or uncoated. The results were presented as the mean ± SD (standard deviation).

**Figure 6:**
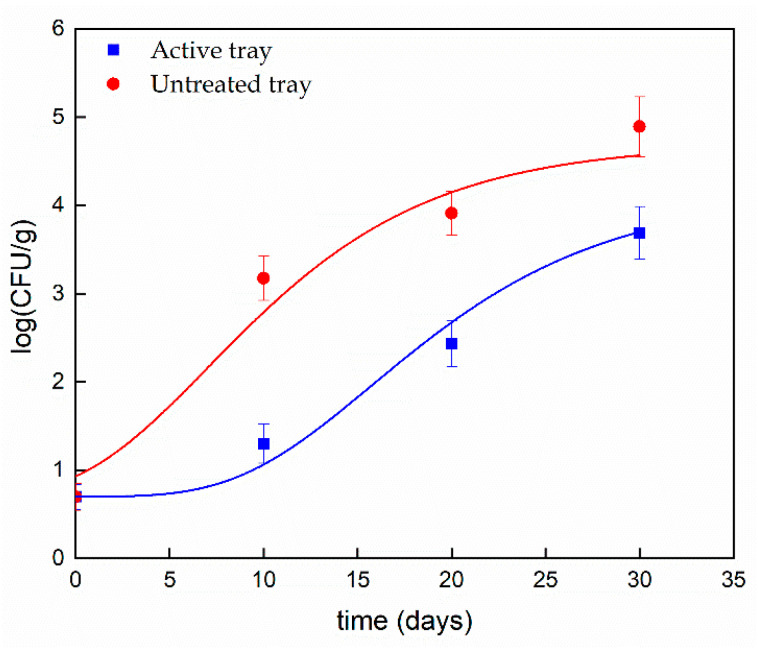
Evolution of molds on packaged pasta during storage time (scatters) and fitting curves (solid lines) obtained from Equation (1).

The microbiological analysis of molds and TBC present in both the pasta packed in the uncoated tray and in pasta packed in the tray treated with the active filler was carried out in triplicate at different times; time zero, after 10 days, 20 days, and 30 days. At all times of analysis, it could be observed that the Log values (CFU/g) in the active tray were lower than those of the uncoated tray, reaching a difference of almost 1–2 logarithmic orders of magnitude. Such results, which were significantly different (*p* < 0.05), highlighted that the active trays containing the LDH-(4-hydroxybenzoate) possessed a strong inhibitory power towards molds and TBC on the considered packaged food. The Gompertz equation was applied to estimate the shelf life (SL) of ready-to-eat pasta by fitting Equation (1) [39]:

(1) $$\log\left(CFU\right)=K+A*\exp\left\{-exp\left\{\left[\left(\mu_{max}*2.7182\right)*\frac{\lambda -t}{A}\right]+1\right\}\right\}$$

where *K* (log(CFU/g)) is the initial level of the bacterial count, *μ_max_* is the maximum growth rate, *λ* is the lag phase (days), *A* is the maximum bacteria growth achieved at the stationary phase, and t is the time (days). After estimating Gompertz’s equation parameters, shelf life was evaluated by using Equation (2):

(2) $$S.L.=\lambda -\frac{A*\left\{ln\left[-ln\left(\frac{\log\left(1\;*\;10^{2}\right)-K}{A}\right)\right]-1\right\}}{\mu_{max}*2.7182}$$

where 1 ∗ 10^2^ is the acceptability limit for the mold evolution of cooked pasta. All analyses were carried out in triplicate. The coefficient of determination (*R*^2^) is reported in Table 4. The media and standard deviations were calculated. The results concerning the mold evolution on packaged pasta are reported in Figure 6.

The evaluated parameters from Equation (2) are reported in Table 4. The statistical analysis showed a significant difference (*p* < 0.05).

Table 4 clearly evidences the differences between the two types of packaging, suggesting that 4-hydroxybenzoate affected the maximum cell growth rate and the cell growth rate in the stationary phase. Moreover, it is worth noting a noticeable increase in lag time, which doubled for the active tray (from 7 days to 14 days) and a halving of *μ_max_* (0.11 and 0.06 days^−1^ for the untreated and the active tray, respectively). As shown in the above table, the active tray slowed the growth of the molds during cooked pasta storage, allowing to extend the shelf life up to about 55 days, noticeably prolonged in comparison with the shelf life of pasta stored in untreated tray (about 21 days). Table 5 shows the microbiological analysis respect to *Enterobacteriaceae,* Lactic Bacteria, and *Pseudomonas* evaluated on the cooked pasta stored either in the active and uncoated trays. It is worth noting that the starting bacteria counts, for all considered strains, are very low. The very low values of bacteria counts, in all cases, remained below 10 CFU/g up 20 days of storage in all packaging. At 30 days it was visible as an increasing 2–3 order of magnitudes for the pasta packaged into untreated trays, and still a value of such strains below 10 CFU/g for the pasta stored in the active trays. The statistical analysis on active and untreated trays showed a significant difference (*p* < 0.05) after 30 days. It follows that the active packaging, was able to prevent the considered bacteria proliferation for this long time.

**Figure 7:**
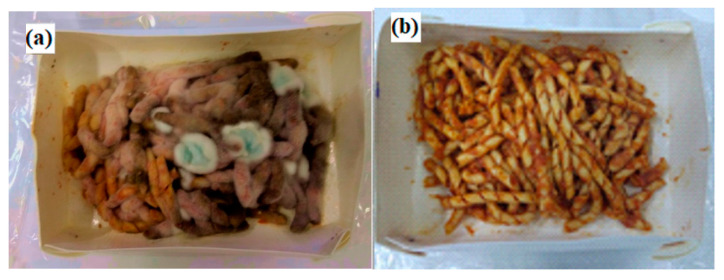
Pictures taken on cooked pasta with tomato sauce stored for two months at 4 °C in: (**a**) an uncoated tray; (**b**) and active tray.

Figure 7 shows, as a visual example, images of pasta stored for two months at 4 °C in both prepared packaging.

After 2 months at 4 °C, molds covered almost the total surface of the pasta stored in the uncoated tray. A very different picture (few molds ≅ 5%) was seen for the pasta packaged in the active packaging, demonstrating the inhibitory effect of fillers towards the proliferation of molds, even after two months of storage.

## 4. Conclusions

Cellulose-based trays coupled with cellulose acetate, coated with an active filler, were prepared and tested as packaging for cooked pasta with tomato sauce, ready to eat. The active coating was based on a food-grade resin filled with layered double hydroxide (LDH) nanofiller hosting 4-hydroxybenzoate, as an antimicrobial, listed in EC-Directive 10/2011/EC of 14 January, 2011. The in-vitro bacterial inhibition on the active trays against *Pseudomonas, Escherichia coli, Salmonella,* and Lactic Bacteria was analyzed, and a significant antibacterial activity was evidenced, in particular respect to *Pseudomonas* and *Salmonella*. Global migration tests on the active trays using acetic acid at 3% (*v*/*v*), ethanol at 50% (*v*/*v*) and vegetable oil, as food simulants, resulted in compliance with the migration limits imposed from EU regulation, demonstrating the suitability of the prepared material for food contact. Ready to eat, freshly cooked pasta with tomato sauce was packaged in the active and uncoated trays, as control, up to 30 days at 4 °C. Organoleptic analyses, mold count, Total mesophilic aerobic counts (TBC), *Enterobacteriaceae,* Lactic Bacteria, and *Pseudomonas* count showed an important antimicrobial activity of 4-hydroxybenzoate for increasing the shelf life of the packaged pasta using a cheap, safe, and easily industrially scalable process.

## Figures and Tables

**Table 1:** Inhibition of *Pseudomonas, Escherichia coli, Salmonella,* and *Lactobacillus* from the active trays, following the directive ISO 22196:2011.

Bacterial Strain	*Pseudomonas aeruginosa* ATCC 15442	*Escherichia coli* ATCC 8739	*Lactobacillus sakei* ATCC 15521	*Salmonella enterica typhimurium* ATCC 14028
Sample size (mm × mm)	50 × 50	40 × 40	50 × 50	50 × 50
Sample thickness (mm)	1.0	0.070	0.1	1.0
Inoculum volume (mL)	0.4	0.4	0.4	0.4
Number of bacteria available in the inoculum	410.000	25.000	35.000	120.000
Uo—Counts bacteria recovered from untreated specimens after inoculation (Log units)	4.4 ± 0.0026 (Log CFU/cm^2^)	4.3 ± 0.0024 (Log CFU/cm^2^)	3.4 ± 0.0021 (Log CFU/cm^2^)	3.9 ± 0.0022 (Log CFU/cm^2^)
Ut—Count of bacteria recovered from non-treated samples after 24 h from inoculation (Log units)	5.3 ± 0.0032 (Log CFU/cm^2^)	5.7 ± 0.0034 (Log CFU/cm^2^)	2.5 ± 0.0014 (Log CFU/cm^2^)	4.3 ± 0.0025 (Log CFU/cm^2^)
At—Count of bacteria recovered from treated samples after 24 h from inoculation (Log units)	n.d. *	4.6 ± 0.0026 (Log CFU/cm^2^)	0.8 ± 0.0011 (Log CFU/cm^2^)	1.6 ± 0.0013 (Log CFU/cm^2^)
Antibacterial activity R = (Ut − Uo) − (At − Uo) (ISO 22196:2011)	>5.3	1.1	1.6	2.6

* Not detectable.

**Table 2:** Overall migration from the active trays, according to UNI EN 1186-1:2003 and UNI EN 1186-9: 2003.

Simulant	B—Acetic Acid at 3% (*v*/*v*)	D1—Ethanol at 50% (*v*/*v*)	D2—Vegetable Oil	Limits
Temperature of the test (°C)	40	40	40	
Contact time (days)	10	10	10	
Overall migration (mg/dm^2^)	7.41 ± 0.03	9.05 ± 0.08	7.72 ± 0.05	10

**Table 3:** Organoleptic analysis at 30 days in uncoated and active trays evaluated according to regulation M.I. 1923A Rev0 2012.

Characteristic Observed	Uncoated Trays	Active Trays
appearance	Water condensation on the external packaging. Presence of visible mold	No water condensation on the external packaging. The appearance of the food typically regular, even if with water droplets on the entire surface of the sample
color	Not regular, with a slightly darker note.	Typical regular.
texture	not regular.	typical regular.
smell	not acceptable due to the presence of degradative notes	typical regular
taste	not acceptable due to the presence of degradative notes	acceptable with the absence of significant degradative notes
visible moulds (%)	≅80	≅5

**Table 4:** Gompertz’s equation parameters evaluated by using Equations (1) and (2).

	Untreated Tray	Active Tray
K	0.70 ± 0.07 ^a^	0.68 ± 0.04 ^a^
A	1.62 ± 0.18 ^a^	1.35 ± 0.20 ^b^
μ_max_ (days ^−1^)	0.11 ± 0.02 ^a^	0.06 ± 0.01 ^b^
λ (days)	7.07 ± 1.16 ^a^	14.78 ± 2.05 ^b^
Shelf life (days)	20.70 ± 1.21 ^a^	54.47 ± 1.35 ^b^
R^2^	0.989	0.990

Means having different superscript lowercase letters for a parameter were significantly different (*p* < 0.05) through the Tukey test.

**Table 5:** Evaluation of *Enterobacteriaceae,* Lactic Bacteria and *Pseudomonas* in the uncoated and active trays.

Time (Days)	Sample	Enterobacteriaceae (CFU/g)	Lactic Bacteria (CFU/g)	*Pseudomonas* spp. (CFU/g)
0	Untreated tray	<10 ^a^	<10 ^a^	<10 ^a^
	Active tray	<10 ^a^	<10 ^a^	<10 ^a^
10	Untreated tray	<10 ^a^	<10 ^a^	<10 ^a^
	Active tray	<10 ^a^	<10 ^a^	<10 ^a^
20	Untreated tray	<10 ^a^	<10 ^a^	<10 ^a^
	Active tray	<10 ^a^	<10 ^a^	<10 ^a^
30	Untreated tray	≅10^3 a^	≅10^3 a^	≅10^2 a^
	Active tray	<10 ^b^	<10 ^b^	<10 ^b^

Means having different superscript lowercase letters for a parameter are significantly different (*p* < 0.05) through the Tukey test.
